# Morphogenesis of the Metanephric Kidney

**DOI:** 10.1100/tsw.2002.854

**Published:** 2002-06-28

**Authors:** Jamie A. Davies

**Affiliations:** Anatomy Building, Edinburgh University Medical School, Teviot Place, Edinburgh, EH8 9AG, UK

**Keywords:** kidney, morphogenesis, nephrogenesis, branching, differentiation, mesenchyme, epithelium, nephron, glomerulus, angiogenesis, vasculogenesis

## Abstract

The metanephric (permanent) kidney of the mouse is an exceptionally well-studied example of organ development. Its morphogenesis begins on the meeting of two tissues, the epithelial ureteric bud and the metanephrogenic mesenchyme; a series of signalling events between these tissues and their successors organizes the organ as it grows and matures. Many of the signals have been identified at the molecular level. They include GDNF, neurturin, persephin, HGF, BMP-2, BMP-7, FGF-10, activin, and TGFβ (all of which control development of the ureteric bud); TGFα, TIMP-2, BMP-4, and BMP7 (all of which control development of the mesenchyme); LIF, FGF-2, TGFβ, Wnt-4, sFrp, Notch, and Jagged (all of which control nephron development); and VEGF (which controls vascularization). Many of these signals are arranged in feedback loops, so that cells entering one developmental pathway signal back to ensure that other cells are more likely to enter alternative pathways, and thus keep the relative proportions and positions of different renal tissues in balance.

## INTRODUCTION TO VERTEBRATE KIDNEYS

The kidneys of vertebrates have a characteristic architecture in which a number of nephrons filter plasma, recover solutes still required by the body, then drain into a common collecting duct. The most primitive type of vertebrate kidney, the pronephros, is functional in early larvae of anamniotes (fish and amphibians). A pronephros consists of one or more nephrons, the proximal ends of which are simple ciliated funnels opening into the coelom and the distal ends of which drain into a common nephric duct that leads caudally to open into the cloaca. There is one

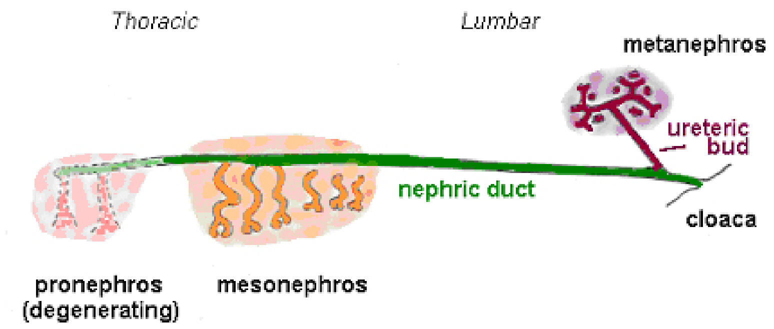

**FIGURE 1.** The three types of vertebrate kidney. The simple pronephros, here shown already degenerating, is the functional excretory organ of aquatic larvae but is quickly replaced by the mesonephros. Anamniotes retain their mesonephroi as excretory organs in the adult, often connecting them (in males) to the testis to provide a route for sperm to leave the body. In amniotes (reptiles, mammals, and birds), a third type of kidney, the metanephros, forms caudal to the mesonephros and the latter organ then either degenerates completely to becomes adapted to form male genital ducts.

pronephros and one nephric duct on each side of the body. As larvae develop, they form a second pair of kidneys, the mesonephroi. These organs follow the same general arrangement of having a series of nephrons that are arranged side-by-side along the nephric duct into which they drain, but the nephrons of mesonephric kidneys begin not as ciliated funnels but rather as proper glomeruli in which blood capillaries and nephric epithelia are in intimate contact. The mesonephros is the adult kidney of agnatha, fish, and amphibians.

When fully terrestrial vertebrates arose about 300 million years ago, they developed a third type of kidney for adult life, the metanephros (Fig. 1). The most obvious differences between metanephroi and mesonephroi are that the metanephroi develop more caudally, and each is arranged around a highly branched rather than a linear collecting duct system, making it a much more compact structure. The nephrons of metanephric kidneys also possess an extra segment, the loop of Henle, which aids urine concentration and thus conservation of water. Although reptiles, mammals, and birds use only metanephric kidneys for excretion in adult life, they still form mesonephroi during embryonic development and subsequently lose them or modify them to serve as part of the male genital duct.

Although the intense recent interest in zebrafish and frog development is beginning to uncover some of the mechanisms of pro- and mesonephric development, most of what we know about how kidneys develop has been discovered in the metanephros, particularly that of the mouse. For this reason, the rest of this mini-review will be concerned only with morphogenesis of the metanephros and, following established medical practice, this mini-review will use the words “kidney” and “renal” to refer only to this organ.

## THE ANATOMY OF METANEPHRIC MORPHOGENESIS

Metanephric morphogenesis in the mouse begins at about 10 days postcoitum, when the caudal end of the nephric duct evaginates to form a tubular outgrowth, the ureteric bud. The ureteric bud grows towards a specialized area of the caudal end of the intermediate mesoderm, called the metanephrogenic mesenchyme. Once the ureteric bud makes contact with and invades the metanephrogenic mesenchyme, it begins to undergo branching morphogenesis to create an extensive tree of tubules that differentiate into mature collecting ducts (Fig. 2). The ‘stalk’ of the

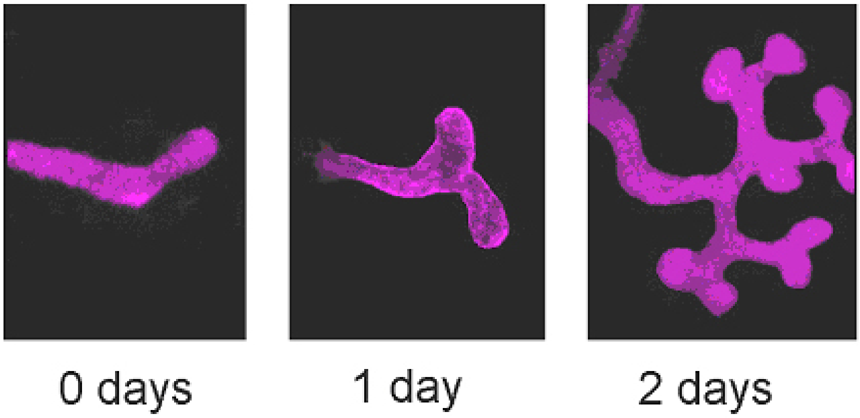

**FIGURE 2**. Branching morphogenesis of the ureteric bud. In these images, kidney rudiments were cultured for 0, 1, or 2 days, fixed and stained for the bud-specific marker Calbindin-D-28k[73]. The mesenchyme that surrounds the bud cannot be seen in these images.

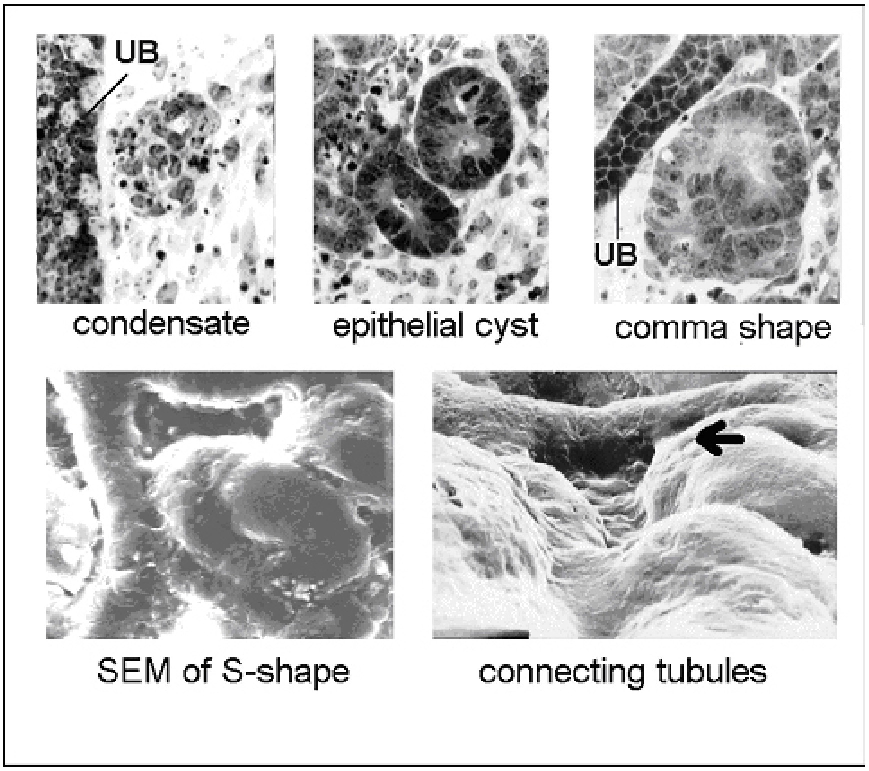

**FIGURE 3**. Stages in nephron development. The first stage is condensation of mesenchyme cells near into a tight aggregate near the ureteric bud ("UB"). The condensed cells then undergo a mesenchymal-to-epithelial transition. These then invaginated to form comma and 'S' shaped tubules. As the proximal slit of the "S" forms the glomerulus (not shown) the distal pole connects to the ureteric bud; the first stages of this are shown in the final electron micrograph. All of these images were obtained from cultured kidney rudiments.

ureteric bud between the mesenchyme and the nephric duct develops into the ureter itself, and the adjacent region of the cloaca later forms the bladder.

As the ureteric bud branches and grows, its tips first induce the mesenchyme to set aside its default ambition to die by apoptosis[1], and they then induce it to condense. Groups of condensed cells then undergo epithelial differentiation to produce small epithelial cysts, which follow a stereotyped series of morphogenetic changes to make comma- and S-shaped tubules (Fig. 3). One cleft of the "S" thins and differentiates into a glomerulus, the cleft being invaded by developing capillary endothelia, while the other end of the "S" fuses to a nearby collecting duct. The regions of the nephron so formed go on to mature into physiologically and anatomically distinct segments, the proximal convoluted tubule, loop of Henle, and distal convoluted tubule. As the complex development of epithelial tubules takes place, the endothelial plumbing of the vascular system has to develop in intimate association with it. Endothelial cells invade the proximal cleft of the S-shaped tubule to create the glomerular tuft, and this capillary complex links to afferent arterioles connected, eventually, to the renal artery, and efferent arterioles which conduct blood to capillaries around more distal parts of the nephron before it is collected by venules.

## SPECIFICATION OF THE METANEPHROGENIC MESENCHYME

Differentiation of the metanephrogenic mesenchyme from the caudal end of the intermediate mesoderm on each side of the embryo is a prerequisite for renal morphogenesis. The process by which metanephrogenic mesenchyme is determined is not yet understood, but mutant mice have implicated a few transcription factors in the process. The homeodomain protein Lim-1, for example, is expressed throughout the intermediate mesoderm and in its absence neither meso- nor metanephric kidneys form[2,3]. The transcriptional activator Eya-1 is also required for differentiation of the caudal end of the intermediate mesoderm into metanephrogenic mesenchyme, and without it this region fails to express markers characteristic of the tissue[4]. The paired domain transcription factor, Pax-2, is expressed in the early development of both meso- and metanephroi, and in its absence neither organ forms[5]. Pax-2 has a very closely related paralogue, Pax-5, because of a gene duplication event about 400 million years ago[6,7]. It is striking that if Pax-5 is placed under a Pax-2 promoter in an attempt to rescue the *pax2-/-* mouse, most tissues, including the mesonephros, develop normally but the metanephros fails to form[8]; this suggests that the metanephros, a 'new' organ just 300 million years old, evolved to use features of Pax-2 acquired after its divergence from Pax-5, whereas more ancient parts of the body plan use only those features of Pax-2 that were present before the Pax 2-5 gene duplication. The winged helix protein Foxc-1 has a role in either setting up or interpreting positional information along the craniocaudal axis of the intermediate mesoderm. In its absence, metanephrogenic mesenchyme does differentiate, but it does so too far craniad that frequently induces the formation of supernumerary ureteric buds and duplicate kidneys[9].

## INDUCTION OF URETERIC BUD EMERGENCE

The ureteric bud seems to emerge from the nephric duct primarily in response to Glial cell-line–derived neurotrophic factor (GDNF), which is secreted by metanephrogenic mesenchyme as soon as it differentiates. The nephric duct expresses the GDNF receptor complex, which consists of the Ret receptor tyrosine kinase and the GRF$\alpha$1 coreceptor, and the whole region of the nephric duct between the mesonephros and the cloaca is capable of producing a ureteric bud in response to a local source of GDNF[10]. GDNF cannot, however, be the only signal capable of eliciting bud emergence because 27% of *gdnf-/-* mice do still manage to produce buds and begin to form small kidneys[11], as do a small proportion of *ret-/-* mice[12,13].

Emergence of the ureteric bud also depends on the presence of a little-understood cytoplasmic protein, formin IV[14,15]. The function of this protein has not yet been elucidated, but in renal epithelial cell lines it is translocated to the nucleus in response to morphogens such as hepatocyte growth factor (HGF)[16]. It may therefore be involved in signal transduction, either of the GDNF signal or some other signalling event.

The nephric duct is not affected only by positive influences, though, and it is surrounded by stromal cells that secrete BMP-4 which antagonizes bud outgrowth[17]. This presumably acts to ensure that the bud is produced only from the region of nephric duct that is right next to the metanephrogenic mesenchyme, and that ectopic buds do not form too early or too cranially. Support for this model comes from *bmp4+/-* heterozygotic mice, which frequently have ureteric buds that emerge from the duct at too high a level and that are often split into two ureters.

## BRANCHING MORPHOGENESIS OF THE URETERIC BUD

Once it has penetrated the metanephrogenic mesenchyme, the ureteric bud is altered by mesenchyme-derived components. This alteration can be seen morphologically by the fact that it begins to branch instead of simply elongating. It can also be seen at a molecular level. Before contact with the mesenchyme, for example, the ureteric bud expresses both Pax-2 and Ret independently of the transcription factor Emx-2, but once it is in the mesenchyme Pax-2 and Ret expression depend on Emx-2 and fail in *emx2-/-* mice[18]. The GDNF-Ret signalling system that was required for emergence of the ureteric bud from the nephric duct is still required for its branching morphogenesis, and depriving cultured kidney rudiments of GDNF signalling using antibodies inhibits branching[19]. Treating them with exogenous GDNF, on the other hand, increases branching and local sources of GDNF attract branches towards themselves[10]. Two other members of the GDNF family of neurotrophins, neurturin and persephin, also encourage ureteric bud branching in culture[20,21]. Of these, neurturin is especially interesting because it is expressed by the bud itself rather than by the mesenchyme, so presumably acts in an autocrine manner.

Hepatocyte growth factor (HGF), which signals via the Met receptor tyrosine kinase, is a morphogen implicated in a number of instances of branching morphogenesis which can even drive branching from simple cysts of epithelial cells in 3-dimensional collagen matrices[22]. HGF is produced by metanephrogenic mesenchyme and is required for collecting duct branching in culture[23,24] but is apparently dispensable *in vivo* as the kidneys of *hgf-/-* mice are normal[25]. Signalling by the unknown ligand of the Ros receptor tyrosine kinase is also required, at least in culture where antisense inhibition of Ros expression results in abnormal bud morphogenesis[26].

All of the positive signals mentioned above act via the Erk MAP-kinase and the PI-3-kinase pathways. Ligands that stimulate signalling via the Smad signal transduction pathway, on the other hand, inhibit branching. The bone morphogenetic protein BMP-2 signals via Smad-1 to inhibit branching[27]. It is produced by groups of mesenchyme cells as they condense together to begin to form a nephron, which they are induced to do by the tips of the growing ureteric bud (see below). The passage of a growing bud tip through an area of mesenchyme therefore has the effect of switching that mesenchyme to being less supportive of new ureteric bud branching, creating a feedback loop that confines branching activity to areas of virgin mesenchyme rather than allowing it to take place in areas already close to a stalk of ureteric bud. BMP-4 is produced by stroma as it differentiates from the mesenchyme, along the maturing ureteric bud/collecting duct stalks. BMP-4 inhibits branching morphogenesis, but can encourage tubule elongation[17]. BMP-7 inhibits branching at high concentrations but seems to facilitate it at low ones[27]. It is produced at moderate levels by mesenchyme and at higher ones by epithelia, so may be another mechanism to prevent the production of new branches in areas already rich in epithelium. Receptors for activins and TGF$\beta$ signal via the Smad 2/3 pathway[28]. Like BMP-4, TGF$\beta$ is produced in the stroma and, at least in culture, exogenous TGF$\beta$ generates abnormally long, unbranched tubules[29]. It is not clear whether it does so by increasing elongation, by preventing branching or by a combination of both.

As well as requiring receptors for growth factors, morphogenesis of the ureteric bud requires receptors and components of the extracellular matrix. Heparan sulphate glycosaminoglycans, carried by membrane-associated proteoglycans such as syndecan, are also required for epithelial growth and branching. Renal morphogenesis is inhibited if heparan is removed in culture[24] or if its synthesis is inhibited in transgenic mice lacking heparan sulphate-2-O-sulphotransferase[30]. Paradoxically, although ureteric bud morphogenesis depends on the presence of matrix components it cannot take place unless some matrix is destroyed in order to create space for the advancing bud tip. Matrix metalloproteinases, especially MMP-9, are critical to this in culture[31] although *mmp9-/-* mice have normal kidneys, suggesting that other MMPs can substitute *in vivo*[32]. Tissue inhibitors of metalloproteinases such as TIMP-2, or pharmacological inhibitors such as ilomastat, antagonize MMP-9 in culture and inhibit bud branching[33].

## RESCUE OF THE MESENCHYME FROM APOPTOSIS

The default fate of the metanephrogenic mesenchyme is apoptosis; isolated mesenchymes die by apoptosis in culture, and also *in vivo* when the ureteric bud fails to form[1]. Mesenchymal cells are diverted from their suicidal yearnings by soluble factors secreted by the ureteric bud. One conventional growth/survival factor that acts this way is TGFα, which is made by the bud[34]; stimulation of the TGFα/EGF receptor with EGF in cultured metanephrogenic mesenchymes promotes their survival[1]. An unconventional one is the protease inhibitor, TIMP-2, which is synthesized by the bud and which promotes mesenchymal survival by a mechanism apparently different from its widely known protease inhibiting activity (a point that can be illustrated by the failure of pharmacological inhibitors of proteases to mimic TIMP-2 activity on mesenchymal survival [33]).

Survival of the existing mesenchyme, though essential, is not enough to support renal development, for the organ also has to grow which involves a continual centrifugal expansion of the organ rudiment to provide a rim of undifferentiated cortical mesenchymal cells into which the tips of the ureteric bud can continue to grow. Known signals that promote proliferation of the mesenchyme are linked tightly to epithelial position and differentiation so that development of the epithelial and mesenchymal compartments of the kidney are kept in balance. BMP-4 induces a strong proliferative response in metanephrogenic mesenchyme, and the main sources of BMP-4 are differentiating nephrons and stroma[17]. The cells that form nephrons and stroma are recruited from the metanephric mesenchyme under the influence of inductive signals from the bud (see below). Loss of mesenchyme to nephric and stromal differentiation therefore stimulates proliferation of the remaining mesenchyme so that the lost cells are replaced, a clever example of feedback. BMP-7, also produced by differentiating nephrons, aids both mesenchymal survival and proliferation and the kidneys of *bmp7-/-* mice have a shrinking, apoptotic population of mesenchymal cells at their cortex where a healthy proliferating population should be; this seriously limits the eventual size of the organs[35]. This effect of BMP-7 may be quite indirect, however, as it also has a strong effect on stroma and therefore on the production of stroma-derived factors (see below)

## INDUCTION OF NEPHRON DIFFERENTIATION

As well as producing factors that ensure mesenchymal survival, the ureteric bud secretes proteins that induce some mesenchymal cells to group together and to differentiate into nephrons. This inductive process is still not well understood, but a few proteins have been identified which, together, seem to be able to reconstitute its activity. The protein FGF-2, produced by the ureteric bud[36], can prevent apoptosis in isolated rat mesenchyme and it also induces upregulation of the transcription factor WT-1. Weeks later, small and incomplete nephrons form in FGF-2-treated mesenchyme[37], though this happens only in rats, not mice. Leukaemia inhibitory factor (LIF)

cooperates with FGF-2 and the combination can induce nephrons within just 7 days in culture[38]. Addition of TGFβ, normally made by stromal cells around the ureteric bud stalks, to this mix causes nephrons to form within 2–3 days, their *in vivo* speed[38]. The ureteric bud also expresses Wnt-11 at the very tips of its branches, exactly where one would expect an inducer to be located. Unfortunately, however, there is no evidence to date to implicate Wnt-11 in nephron induction and other cells engineered to express Wnt-11 do not become surrogate inducers[39].

Once the mesenchyme cells have been induced, they alter their expression first of transcription factors and then of morphoregulatory molecules. One of the earliest transcription factors to be upregulated is WT-1, a zinc finger protein first identified in a search for the genetic basis of chromosomal deletions that predispose to Wilms' Tumour, a congenital renal neoplasm[40]. Without WT-1, metanephrogenic mesenchyme is incapable of differentiating into nephrons, and it eventually dies[41]. The precise function of WT-1 in renal development remains unclear, but cell-line assays have implicated one of its splice forms in regulation of Pax-2, syndecan, and E-cadherin[42], all genes that are important in later stages of nephron development (see below).

After upregulation of WT-1, mesenchymal cells condense around the ureteric bud and then condense more tightly into approximately spherical aggregates that undergo mesenchymal to epithelial transition. During condensation, the cells express Pax-2, which is absolutely required for subsequent development[43]. The cells also express Hox-a11 and Hox-d11; mice lacking either one of these have normal kidneys but the kidneys of the double knockout are severely hypoplastic[44]. Condensation of cells into a tight aggregate is accompanied by expression of the signalling protein Wnt-4. This protein is required for the aggregates to go on to become truly epithelial, and in *wnt4-/-* mice this process fails[45]. Wnt-4 acts in an autocrine loop, driving its own expression and it is possible that the Wnt-4 positive feedback system is used by condensing cells to assess their population size; if they find themselves surrounded by cells expressing Wnt 4 they themselves express it, and once the level is high enough they can go on to differentiate, secure in the knowledge that they must be in a sufficiently large group of similarly committed cells for nephron formation to be possible[46]. This model for Wnt-4 action may explain the results of Davies and Garrod[47], who showed that lithium ions can induce nephrons in isolated metanephrogenic mesenchyme but that the nephrons formed were abnormally small and developed abnormally quickly. Lithium acts by mimicking Wnt signalling[48], so presenting a false Wnt signal using lithium would cause the cells to act as if they were in a larger condensate than they really were, and they would differentiate prematurely.

The Wnt-4 autocrine loop must be prevented from recruiting all cells into the condensate, though, because the population of metanephrogenic mesenchyme must be maintained. A negative influence is provided by sFRP-1, a secreted member of the Frizzled family of Wnt receptors that competes with and therefore inhibits Frizzled itself. sFRP-1 is produced naturally by stroma just inside the kidney capsule at the very outside of the organ, and experimental application of it to the whole kidney phenocopies the *wnt4-/-* phenotype[49]. Presumably, *in vivo* sFRP diffusing centripetally from the subcapsular stroma antagonizes Wnt-4 diffusing centrifugally from the condensing cells and maintains a band of proliferating mesenchyme that is safe from being drawn into the condensates. To make this system more complicated, sFRP-2, another secreted Frizzled-like protein that antagonizes sFRP-1, is produced by condensed cells that are already unregulating Wnt-4. These cells are therefore able to bind Wnt-4 unimpeded by sFRP-1, so the system acts as a latch, committing cells to their fate and avoiding the possibility of them being trapped in fluctuating states of differentiation as the local concentrations of Wnt-4 vary stochastically.

Once committed to epitheliogenesis, condensed cells synthesize epithelial-type cadherins such as E-cadherin, K-cadherin, cadherin 6, cadherin 11, desmogleins and desmocollins, tight junctional components such as ZO1, and gap junction connexins[47,50,51,52,53]. Internally, they replace their vimentin cytoskeleton with one based on cytokeratins[47,52] and they produce a normal basement membrane that includes collagen IV and laminin-α1[54,55]. As well as being markers for the epithelial state, some of these molecules are required for epithelia to form — interfering with cadherin 6, for example, blocks epitheliogenesis in culture and delays nephrogenesis *in vivo*[51].

## NEPHRON MATURATION

The processes described under the last heading took us from a dispersed mesenchyme to a small epithelial cyst; an eventful journey in developmental terms, but a long way still from the convoluted, regionally differentiated nephrons of a mature kidney. The regional specification of the different nephron segments begins around the time of epithelialization, and by the time the nephron has invaginated to acquire its "S" shape, one pole of the "S" expresses WT-1 at very high levels. This is the presumptive glomerulus, and the WT-1 expressed there inhibits the expression of Pax-2[56]. If Pax-2 is expressed in transgenic mice under the control of a promoter that is not shut down by WT-1, it remains active throughout the nephron and formation of a glomerulus is aborted[57]. It is interesting that WT-1 seems to be essential for several different events at different stages of renal development. What is more, different splice variants of WT-1 have subtly different functions in nephron maturation; mutant mice that can form only the –KTS splice form of WT-1 make glomeruli in which podocytes fail to mature and to produce foot processes, while mutant mice that can form only the +KTS splice form make very few glomeruli, and those that form fail to produce characteristic markers such as synaptopodin and α3-integrin[58]. These different products of the WT-1 gene must therefore each be essential in their own way to glomerular maturation. The lim-box protein Lmx-1b is expressed in a similar pattern to WT-1, and *lmx1b-/-* mice fail to form a proper glomerular basement membrane, the presence of which is important to the proper filtration of plasma[59]. The cell surface–signalling molecule Notch-2 is expressed by developing nephrons, and the glomeruli of mice homozygous for a hypomorphic mutation in *notch2* fail to mature properly. Heterozygotes are normal, but interestingly double heterozygotes for *notch2* and *jagged1*, which encodes a potential Notch-2 ligand, show the same failure of nephron maturation. This suggests (but does not prove) that Jagged-1 may be the ligand for Notch-2 in this system[60].

The involvement of Notch proteins to control glomerular differentiation in mammals is reminiscent of its use in the development of the Malpighian tubules of *Drosophila*[61]. The embryonic origins of excretory organs in vertebrates and insects are very different (mesodermal origin in vertebrates, endodermal in insects) but this is no longer a barrier to considering that developmental processes may be homologous; the homology between insect and mammalian eyes, illustrated by the activity of Pax-6, bears testimony to that[62]. Mere involvement of one similar gene is not in itself a compelling argument for homology, of course, but it does suggest a fruitful line of enquiry.

Differentiation of the remaining segments of the nephron is understood even less than that of the glomerulus. The nuclear factor HNF-1 is expressed in all regions except the glomerulus, and in the absence of this protein, cells fail to express their usual channels and the nephrons are incapable of concentrating urine properly[63].

## FORMATION OF THE BLOOD SYSTEM

The baroque complexity of epithelial plumbing in the kidney is made even more complicated by the addition of the vascular system which, as well as serving the metabolic needs of the kidney, makes a number of special appositions to each nephron in order to shed filtrate and then to reabsorb selected solutes. The best known 'connection' between the vascular system and the

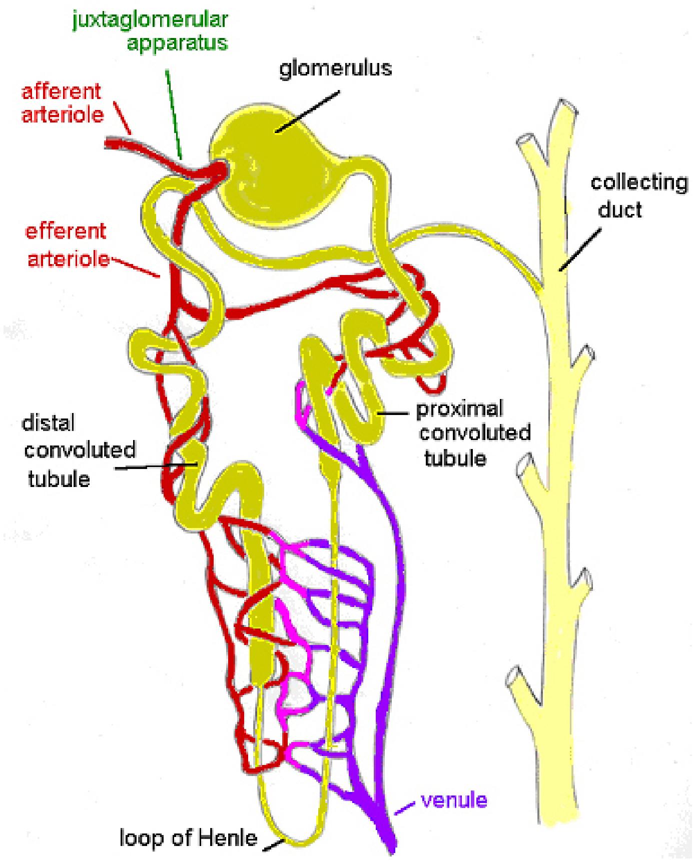

**FIGURE 4.** The blood supply of a mature nephron**.** The microvasculature interacts with the nephron several times, first at the juxtaglomerular apparatus, where the ascending distal tubule interacts with vessels entering the glomerulus, then inside the glomerulus itself, then along the nephron where solutes are passed from the urinary space back to the blood.

nephron is the glomerulus, in which a tight knot of capillary endothelium, specialized for filtration, rests against the podocytes (very specialized glomerular epithelium) of the nephron and water and small solutes flow from the blood vessel across filtration layers into the urinary space. Where the afferent and efferent arterioles lead into the glomerulus, they make a close contact with a specialized area of the distal convoluted tubule called the macula densa, and at this point the walls of the afferent arteriole differentiate to form the renin-secreting juxtaglomerular apparatus. The vasculature then winds closely about the loop of Henle, to form the *vasa recta* (Fig. 4).

Blood vessels in the kidney seem to form by both angiogenesis and vasculogenesis. Early experiments, in which mouse kidneys were grafted on to avian chorioallantoic membrane and became organotypically vascularized, suggested that vascularization normally took place by angiogenesis; after all, all of the correct guidance cues must have been present[64]. One of these guidance cues has been identified as vascular endothelial growth factor (VEGF), which is produced by glomerular epithelium[65]. Anti-VEGF antibodies inhibit glomerular vascularization in culture[66]. Studies of the expression of endothelial markers, such as the Flk-1 VEGF receptor protein, have recently shown that endothelial progenitors seem to be dispersed amongst, or to differentiate from, the metanephrogenic mesenchyme from its earliest stages[67]. In low oxygen environments, these will produce renal vasculature even in the absence of exogenous sources of endothelia (A.S. Woolf, personal communication). Developing renal endothelia also express the Tie-1 and Tie-2 receptor tyrosine kinases, and these too are necessary for the formation of a normal blood supply[68].

## DIFFERENTIATION OF THE STROMA

As well as giving rise to nephrons, the metanephrogenic mesenchyme produces the stroma of the adult kidney. Cells following the stroma fate retain their mesenchymal character (expression of vimentin, etc.) and begin to express proteins such as the transcription factor Fox-d1 and growth factors mentioned earlier such as TGFβ[69,70]. Correct stromal differentiation is vital for normal development of the epithelial compartment of the kidney, and in transgenic mice, such as *foxd1-/-* that have lost components essential for stromal maturation, nephrogenesis aborts and what remains of the kidney fills with large aggregates of disorganized mesenchyme[69]. Stromal cells also carry receptors for retinoic acid, and in the absence of this molecule the ureteric bud fails to maintain expression of Ret[71,72]; this argues for the existence of an unidentified factor that is produced in the stroma only in the presence of retinoic acid, and that then controls Ret expression in the bud.

The balance between stromogenic and epitheliogenic fates in the metanephric mesenchyme is controlled by signals from other compartments of the kidney. Prominent among these signals is BMP-7, which is produced by nephron and bud epithelia. In culture, exogenous BMP-7 causes the cells of isolated metanephric mesenchymes to differentiate into stroma rather than nephrons[73], and *in vivo* it is probably used to ensure that any new epithelia are able to surround themselves with supportive stroma, and that the amount of stromal differentiation is commensurate with the amount of epithelium that requires support.

## THE COORDINATION PROBLEM

To recap for a moment, the developing kidney consists of at least five types of tissue: undifferentiated mesenchyme, ureteric bud, developing nephrons, developing vasculature, and developing stroma (there are also nerves, but this review ignores them because rather little is known about their development). These tissues exist in intimate relation to each other, and normal renal morphogenesis depends on precise balance and relative location of each type of tissue. It will be obvious from the above account that none of these tissues is autonomous, and that every one depends for its development on signals from at least one other tissue type, usually more. In this section of the review I will repeat some of the information already presented, but will organize it not to emphasize the separateness of the anatomical compartments but rather the manner in which their interactions achieve the appropriate balance of fates and locations. I should emphasize that the model presented below is probably incomplete, and while it is compatible with the known expressions and activities of the molecules concerned, most of the feedback loops I propose have not yet been tested directly.

We shall begin near the cortex of a developing kidney, at any time beyond very early development and before development ceases (for mouse, this would be about embryonic day 14 to postnatal day 2). Just under the kidney capsule and its associated layer of stroma is a rind of undifferentiated metanephrogenic mesenchyme. This rind maintains itself by proliferation, in the face of continual losses of cells from its medullary surface to epitheliogenic and stromogenic differentiation. These differentiating cells produce mitogens such as BMP-4 and BMP-7 that induce the mesenchyme they have left behind to proliferate and replace them. Thus the population is maintained. Cells that have left the rind of proliferating mesenchyme to differentiate can follow either nephrogenic or stromogenic fates. The balance between these fates is controlled at least in part by BMP-7, produced by cells that have followed the nephrogenic pathway, so that levels of nephrogenic development are always balanced by appropriate stromogenic development. Whether they follow stromogenic or nephrogenic pathways, cells leaving the proliferating mesenchyme produce signals (BMP2, TGFβ, etc.) to deter the ureteric bud from producing a new towards them, and therefore keep its explorations focussed on the virgin territory of the everexpanding cortex. These control pathways related above have one feature in common, i.e., negative feedback is achieved by a developing cell producing signals that act on the population the cell has just left, to encourage the remaining cells of that population to do the opposite to what that developing cell did.

Vascular development is driven by local signals from the developing nephrons, and therefore automatically keeps pace with the growth of this compartment of the kidney, and it is also encouraged by low oxygen. Our current level of knowledge suggests that the development of the ureteric bud, nephrons, and stroma are sovereign over that of the vasculature, in the sense that endothelial development depends critically on signals developed by the nephron (VEGF, etc.) but avascular nephrons and the collecting duct will develop properly in culture in the absence of endothelia. But these observations are made in culture, and doubtless in a kidney developing *in vivo*, development would be inhibited in any areas seriously short of nutrients and oxygen, so that growth of the epithelial components would not be allowed to outpace that of the vasculature to too great an extent.

Most research done so far into the molecular cell biology of renal development has centered on the discovery of new signals and the characterization of gene expression, and of the phenotypes of single gene mutations. This will no doubt continue to be a fruitful enquiry for years to come – I know of nobody so optimistic that they believe that all of the important signals have already been identified. There is, however, a pressing need to begin studying how all of these signals work together, and to test models, such as those presented above that explain how different aspects of development are coordinated. Only when these aspects are better understood will it be easy to explain the vast range of congenital abnormalities see in the urinary system. Understanding large-scale coordination during organogenesis will also be very important to the developing field of tissue engineering.

This mini-review has concentrated on the aspects of renal development that have been most closely studied and which are best understood. These are still isolated areas of illumination amongst much murk and mystery, and many of the obvious questions about kidney development remain completely unanswered. Examples of such basic questions include "from what did vertebrate kidneys evolve?", "why does the mammalian mesonephros regress?", "why are kidneys of different mammals different shapes?", and "why does the kidney eventually stop growing?". There is still much to be done.

## RELEVANT DATABASE

Kidney development is served by one of the earliest developmental databases to appear on the Internet (1993, but updated continuously). The URL is http://golgi.ana.ed.ac.uk/kidhome.html
